# Adding Assessment of Home Environmental Health Hazards to Home- and Community-Based Service Models

**DOI:** 10.1093/geroni/igaf122.1882

**Published:** 2025-12-31

**Authors:** Steven Albert, Jenn McCartney, Joey Engelmeier, Jemima Ohwobete, James Fabisiak, Sarah Haig, Pamela Toto

**Affiliations:** University of Pittsburgh, Pittsburgh, Pennsylvania, United States; University of Pittsburgh, Pittsburgh, Pennsylvania, United States; University of Pittsburgh, Pittsburgh, Pennsylvania, United States; University of Pittsburgh, Pittsburgh, Pennsylvania, United States; University of Pittsburgh, Pittsburgh, Pennsylvania, United States; University of Pittsburgh, Pittsburgh, Pennsylvania, United States; University of Pittsburgh, Pittsburgh, Pennsylvania, United States

## Abstract

Aging in place refers to an older adult’s ability to safely and independently carry out their desired, necessary, and expected daily activities within their home and community. Home- and community-based services (HCBS) support aging in place by developing care plans based on assessment of disability and the safety and accessibility of homes, but these assessments mostly ignore environmental health hazards, such as poor air quality (PM2.5, CO2, radon) and mold, which are commonly reported by residents. To address this problem, the University of Pittsburgh’s Healthy Home Lab (HHL) developed an air quality assessment and mitigation program, the Healthy Home Assessment Tool (H-HAT), in partnership with four regional HCBS agencies. H-HAT includes assessor observations, resident appraisals, consumer-grade indoor air quality monitoring, pathogen and mold analysis (DNA species extracted from dust). In a pilot with 43 low-income older adult (median age 75) households, 23% reported mold odor, and water damage was observed in 48.7%. While EPA standards for indoor air quality are evolving, almost all homes had PM2.5 values over 9 ug/m3. Fungal species with potential clinical significance were also identified. Care managers at the agencies received a packet reporting H-HAT results, which they discussed with residents. The care managers found the reports useful, which in some cases led them to alter care plans. Residents received resources for mitigation as well as an air purifier as part of the program. Follow-up assessments will determine success for mitigation and help in the design of wider dissemination.

